# Sugammadex for preventIoN oF pOst-operative pulmoNary complIcAtions (SINFONIA): protocol for an embedded mixed-methods process evaluation

**DOI:** 10.1016/j.bjao.2026.100856

**Published:** 2026-09-02

**Authors:** Charlotte Dunn, Bronwen Connolly, Bronagh Blackwood, Jonathan A. Silversides, Lydia M. Emerson, SINFONIA investigators, Louise Savic, Louise Hiller, Katie Booth, Jennifer Dorey, Richard Smithson, Samuel Frempong, Ramani Moonesinghe, Benedict Creagh-Brown, Rebecca Kandiyali, Joyce Yeung, Rupert Pearse

**Affiliations:** 5 Leeds Teaching Hospitals National, Health Service Trust, Leeds, UK; 6 Warwick Clinical Trials Unit, University of Warwick, Coventry, UK; 7 Patient and Public Involvement Representative, London, UK; 8 Patient and Public Involvement Representative, Belfast, UK; 9 University College London, London, UK; 10 University of Surrey, Guildford, UK; 11 Queen Mary University of London, London, UK; 1 Wellcome-Wolfson Institute for Experimental Medicine, Queen's University Belfast, Belfast, UK; 2 Department of Physiotherapy, The University of Melbourne, Grattan Street, Parkville, Melbourne, Australia; 3 Division of Anaesthetics, Critical Care, Theatres and Sterile Services, Belfast Health and Social Care Trust, Belfast, UK; 4 The Medical School, Department of Health and Community Sciences, University of Exeter, Exeter, UK

**Keywords:** anaesthesia, logic model, neostigmine, POETIC framework, process evaluation, sugammadex

## Abstract

**Background:**

The Sugammadex for preventIoN oF pOst-operative pulmoNary complIcAtions (SINFONIA) trial is a multi-centre, pragmatic, randomised superiority trial comparing sugammadex with neostigmine to reverse neuromuscular block after major abdominal or thoracic surgery. Neuromuscular blocking agents are routinely administered during general anaesthesia to facilitate intubation, ventilation, and surgical access. Residual neuromuscular block remains common despite reversal and is associated with increased risk of post-operative pulmonary complications. Sugammadex reduces residual paralysis compared with neostigmine, but its effect on patient-centred outcomes remains uncertain, and clinical equipoise persists. As intervention delivery is embedded within complex intra-operative decision-making, variation in implementation may influence trial outcomes. An embedded process evaluation will characterise intervention delivery and identify contextual and organisational factors influencing implementation across sites. This protocol describes the rationale, aims, and methods of the SINFONIA process evaluation.

**Methods:**

This embedded mixed-methods process evaluation is informed by the PrOcess Evaluation of Trials In Critical care (POETIC) framework, examining Context, Fidelity, Dose, Reach, and Quality of Delivery. A logic model was developed, describing relationships between contextual factors, intervention delivery and trial outcomes. An explanatory sequential design will first analyse quantitative data describing intervention delivery, followed by qualitative data explaining observed variation. Data will be collected via electronic survey and semi-structured interviews, with findings integrated with the main trial results.

**Discussion:**

This embedded process evaluation will characterise SINFONIA intervention delivery across sites, explain how contextual factors shape implementation, and support interpretation of trial findings through integrated process evaluation evidence.

**Clinical trial registration:**

ISRCTN.com ISRCTN15109717. Registered on 21 December 2022.

Randomised controlled trials assume that interventions are implemented and delivered consistently within and between sites. In practice, however, intervention delivery may vary because of organisational and participant-related factors, influencing whether an intervention is delivered, how it is delivered, and how consistently it is delivered. Without understanding these influences, interpretation of trial findings, whether positive, neutral or negative, may be limited.^1^^,^^2^ As outlined in the UK Medical Research Council guidance, process evaluations can be used to ‘assess fidelity and quality of implementation, clarify causal mechanisms and identify contextual factors associated with variation in outcomes’. Additionally, process evaluations can inform decision-making about the transferability of RCT findings and how this may influence implementation approaches for evaluated interventions, including potential barriers or facilitators.^1^ To date, relatively few process evaluations have been conducted alongside anaesthesia and peri-operative medicine trials.^3^ This may reflect an assumption that the intervention is simple, and therefore that a process evaluation is not required. However, there are increasing calls for the integration of process evaluations alongside outcome evaluations in complex healthcare trials.^1^^,^^4^^,^^5^

The Sugammadex for preventIoN oF pOst-operative pulmoNary complIcAtions (SINFONIA) trial is a multi-centre, pragmatic, randomised superiority trial comparing sugammadex with neostigmine for reversal of neuromuscular blocking agents (NMBAs) after major abdominal or thoracic surgery.^6^ Neuromuscular blocking agents are routinely administered during general anaesthesia to facilitate intubation, ventilation, and surgical access.^7^ At the end of surgery, reversal agents are used to restore neuromuscular function; however, residual neuromuscular block remains common and is associated with an increased risk of post-operative pulmonary complications.^8^ Sugammadex has been shown to reduce residual paralysis,^9^ compared with neostigmine, but its effectiveness in improving patient-centred outcomes remains uncertain, and clinical equipoise persists.

Although SINFONIA compares two pharmacological interventions, delivery of the allocated reversal strategy is embedded within complex intra-operative decision-making. Administration and dose selection occur in real time and are influenced by anaesthetist judgement, usual practice, perceived patient risk, and organisational factors such as theatre workflow and local drug access. As a result, the intervention is not fully standardised. Variation in implementation and delivery within and between sites, as well as intra-individual practice, is likely. This variation may influence trial outcomes and their interpretation, particularly in a pragmatic, multi-centre design.

Process evaluations are particularly valuable in such settings, where interventions are implemented across diverse clinical environments and may be subject to variation in delivery.^3^^,^^10^ In SINFONIA, an embedded process evaluation will characterise intervention delivery and identify the organisational and participant-related factors influencing implementation and delivery across sites.

This process evaluation is informed by the PrOcess Evaluation of Trials In Critical care (POETIC) framework,^11^ which provides a structured approach to examining intervention delivery and contextual influences. Although originally developed for critical care trials, POETIC addresses challenges also present in peri-operative anaesthesia, including time-pressured decision-making, variability in clinical practice, and organisational constraints.

If either sugammadex or neostigmine is found to be superior, findings from the process evaluation will help identify the conditions required for successful implementation and inform the transferability of results across settings. Conversely, if no difference is observed, the process evaluation will support interpretation by determining whether findings reflect a true lack of effectiveness or variation in intervention delivery.

## Conceptual framework

This process evaluation is guided by the POETIC framework, which provides a structured approach to examining how trials and interventions are implemented and delivered, and how contextual factors influence variation in delivery and outcomes.

In this process evaluation, four primary domains—Context, Fidelity, Dose and Reach—will be examined directly through empirical data collection. Within this study, Fidelity refers to whether the allocated reversal agent is administered as randomised. Dose refers to how the reversal strategy is delivered in practice, including variation in dose selection. Reach refers to the proportion of eligible patients who receive the randomised intervention. Context encompasses organisational and participant-related factors influencing delivery, including unit culture, organisational structure, resources, usual practice, and attitudes and perceptions.

Quality of Delivery is conceptualised as an integrative construct reflecting the combined influence of Context, Fidelity, Dose and Reach on overall intervention delivery. Rather than being measured as a standalone domain, it will be derived during analysis by integrating quantitative site-level delivery indicators with qualitative data on contextual influences. This will enable intervention delivery to be described as consistent, variable or limited across and within sites, supporting interpretation of variation in delivery and the main trial findings.

The POETIC framework is applied prospectively to guide data collection, analysis and integration. Given the intra-operative nature of the intervention, particular emphasis is placed on contextual factors relating to the deliverer and clinical environment, including anaesthetist decision-making, usual practice, and perceptions of risk and benefit. Patient-related factors will also be explored, particularly in relation to trial participation and consent.

## Aims

The aim of this process evaluation is to characterise delivery of the SINFONIA trial intervention, identify contextual factors influencing implementation, explore trial processes, and support interpretation of the trial findings. The specific objectives are to (1) assess Fidelity by examining whether the allocated reversal agent is administered as randomised; (2) describe Dose by examining how the reversal strategy is delivered in practice, including variation in dose selection; (3) assess Reach by examining the proportion of eligible patients who receive the randomised intervention delivery; (4) explore Context by identifying organisational and participant-related factors influencing delivery, including unit culture, organisational structure, resources, usual practice, and attitudes and perceptions; and (5) derive Quality of Delivery through integrated interpretation of Fidelity, Dose, Reach and Context.

## Design

This study is an embedded mixed-methods process evaluation conducted alongside the SINFONIA randomised controlled trial. It adopts an explanatory sequential design, in which quantitative data describing patterns of intervention delivery are collected and analysed first, followed by qualitative data collection to explore and explain observed variation.

Quantitative data will be used to describe site-level patterns of Fidelity, Dose and Reach. Qualitative data will then be used to explore contextual influences on intervention delivery, including organisational and participant-related factors. Quantitative and qualitative findings will be integrated during analysis to provide a comprehensive understanding of how the intervention was delivered, why delivery varied within and between sites, and how this variation may inform interpretation of the main trial findings.

### Logic model

**Fig 1:**
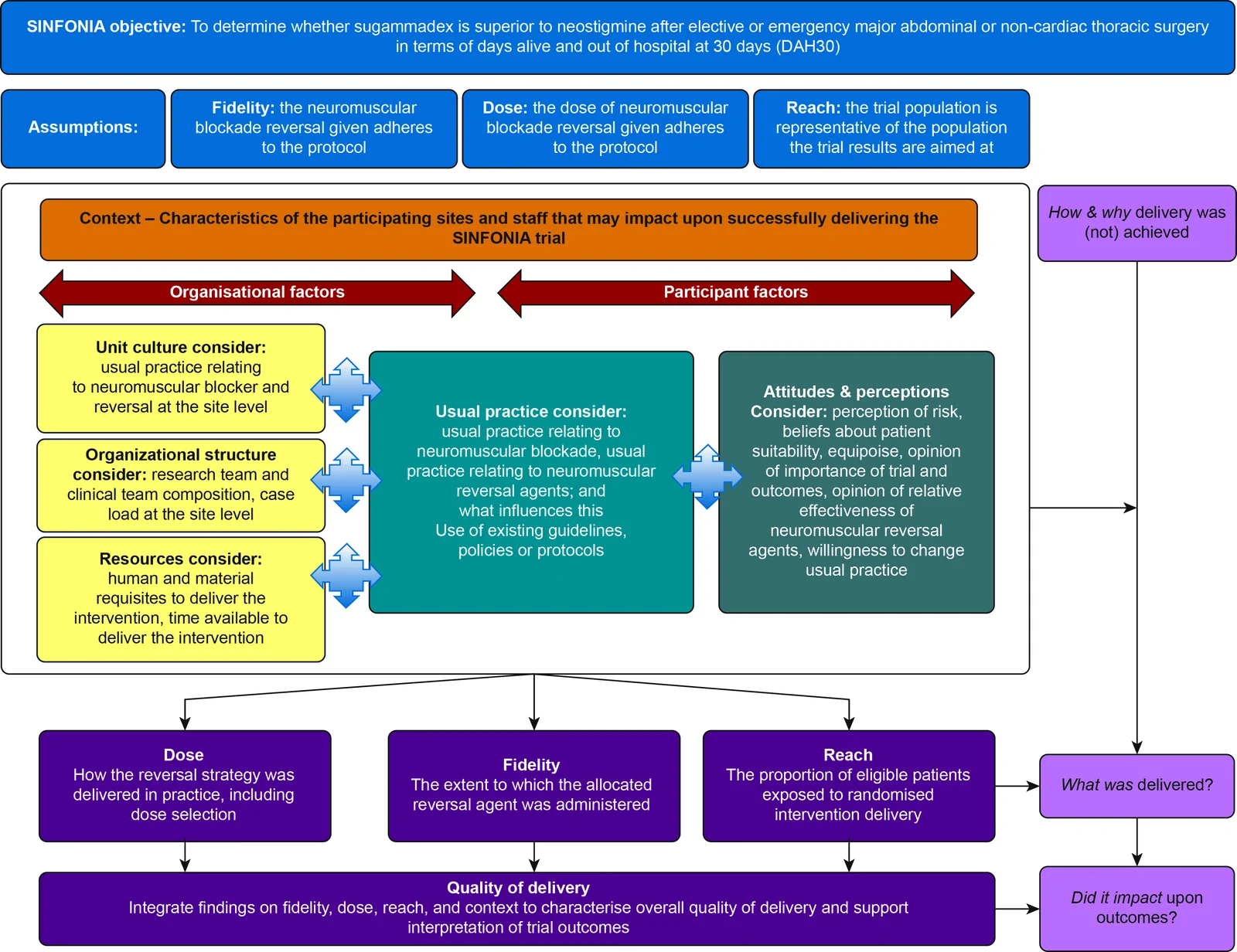
Logic model.

A logic model (Fig. 1) was developed prospectively to describe the assumed relationships between contextual factors, intervention delivery, and trial outcomes. The model was informed by the SINFONIA trial protocol and the POETIC framework domains and was refined through discussion within the trial management and process evaluation teams.

### Process evaluation data collection

Quantitative data will be used to describe patterns of intervention delivery, including Fidelity, Dose and Reach, using routine trial data and screening and recruitment logs. These data will be analysed at site level to identify variation in delivery.

Qualitative data will then be collected to explore contextual influences on delivery, including organisational and participant-related factors. Data will be collected using electronic surveys and semi-structured interviews with anaesthetists and research staff.

Findings from quantitative analyses will inform purposive sampling for qualitative interviews, enabling exploration of sites with differing patterns of delivery (for example, variation in recruitment, protocol adherence, or dose selection). This approach allows qualitative data to explain observed variation in quantitative delivery measures.

### Routine trial data

Quantitative data derived from routine trial datasets will be used to characterise patterns of intervention delivery and to inform sampling for qualitative interviews. These data will include screening and recruitment logs, protocol deviations, and site-level measures of intervention delivery.

Site-level variation in Fidelity, Dose and Reach will be examined descriptively to identify differences in delivery patterns within and between sites. These data will also be used to categorise sites according to delivery characteristics (for example, recruitment rates and protocol adherence), which will inform purposive sampling for the qualitative component.

### Electronic survey

An electronic survey will be used to capture key concepts aligned with the process evaluation objectives and POETIC domains.

Survey participants will include anaesthetists and research staff involved in intervention delivery, recruitment, or trial implementation at participating sites. The survey will be distributed via site principal investigators, who will be asked to complete the survey and to circulate it to up to three anaesthetists most recently involved in delivery of the intervention, as identified by the trial management team. The survey will also be distributed to research staff involved in trial delivery at each site.

Survey items will be developed by the process evaluation research team and informed by the logic model and existing literature. The survey will include both closed-ended questions and free-text responses and will explore intervention-related factors influencing successful implementation and delivery, as well as trial-related factors such as recruitment and Reach. Additional questions for anaesthetists will explore usual practice relating to neuromuscular block and reversal strategies.

The survey will be distributed electronically via REDCap. Participants will receive an invitation containing study information, and informed consent will be obtained electronically before participation. Completion is expected to take approximately 10 minutes. Response rates will be monitored at site level, and reminders will be issued to maximise representation across sites and roles.

### Interviews

Semi-structured interviews will be conducted to explore participants’ experiences of trial delivery, including factors perceived to impact trial delivery, work processes, and engagement with the intervention.

At the end of the survey, participants will be invited to indicate their willingness to take part in a follow-up interview by providing their contact details. This constitutes an initial self-selection (volunteer) sampling stage, generating a pool of participants with direct experience of trial delivery. From this pool, participants will be selected using purposive sampling, informed by routine trial and survey data. Using routine data, sites will be characterised according to key indicators of intervention delivery, such as recruitment rates and protocol adherence. Using survey data, anaesthetists will be characterised according to whether their preferred reversal agent is sugammadex, neostigmine, or no preferred reversal agent. Participants will then be sampled to ensure representation across a range of these delivery contexts and performance profiles. A maximum variation approach will be used to capture diverse perspectives across sites with differing levels of recruitment, protocol adherence and preferred reversal agent practice.

Sampling will be judged sufficient when perspectives have been obtained from site principal investigators, anaesthetists and research coordinators across each sampling category, and when the data provide adequate depth and diversity.

The interview topic guide will be informed by the logic model and POETIC domains, covering contextual influences, including unit culture, organisational structure, resources, usual practice, attitudes and perceptions, as well as Fidelity, Dose, and Reach.

All interviews will be conducted remotely using Microsoft Teams, recorded with consent, and transcribed verbatim. A sample of transcripts will be checked by a second researcher to ensure accuracy. Interviews are expected to last 45-60 minutes.

### Data analysis

Analysis will follow a mixed-methods approach, with quantitative and qualitative data analysed separately and then integrated to support interpretation of intervention delivery. The purpose of this approach is to combine measurement of variation in delivery with in-depth exploration of the mechanisms underlying that variation, providing a more comprehensive understanding than either method alone.

Analysis will be structured using the POETIC framework domains (Context, Fidelity, Dose, Reach), with Quality of Delivery conceptualised as an integrative construct derived from these domains. The process evaluation will be conducted and analysed independently of the trial outcome analysis to minimise the risk of bias in interpretation.

### Quantitative analysis

Quantitative analysis will be used to describe patterns of intervention delivery across sites using routine trial data.

**Fidelity** will be assessed by comparing the allocated reversal agent with the agent administered to identify non-adherence and crossover between trial arms.

**Dose** will be assessed by comparing the administered dose with the protocol-specified dose range based on patient weight, to identify variation in dosing and deviations from protocol.

**Reach** will be assessed by comparing the number of eligible patients screened with the number recruited at each site, to describe variation in recruitment and exposure to the intervention.

All quantitative measures will be summarised using descriptive statistics at site and overall levels. These data will be used to characterise variation in intervention delivery within and between sites and to inform qualitative sampling.

### Qualitative analysis

Qualitative data from survey free-text responses and semi-structured interviews will be analysed using framework analysis,^12^ a systematic and transparent approach to qualitative analysis widely used in applied health research, particularly suited to studies with predefined objectives, multidisciplinary teams, and comparative analysis across cases. Analysis will follow an iterative process. Interviews will be transcribed verbatim, and researchers will familiarise themselves with the data through repeated reading of transcripts and survey responses.

An initial analytical framework will then be developed deductively, informed by the POETIC domains and the logic model. Data will be organised within this framework to enable systematic comparison across participants and sites, supporting examination of how intervention delivery is shaped by organisational and participant-related factors.

In parallel, inductive analysis will be undertaken to identify themes not captured within the predefined framework. The analytical framework will be refined iteratively to incorporate these emerging insights. Analysis will be conducted collaboratively by the research team, with regular discussion to ensure consistency, resolve differences in interpretation, and enhance analytical rigour. Mapping and interpretation will involve synthesising findings across the analytical framework to develop explanatory accounts of how and why variation in intervention delivery occurred, including how contextual factors influence Fidelity, Dose, and Reach.

### Integration of quantitative and qualitative data

Quantitative and qualitative data will be integrated during the interpretation phase to provide a comprehensive understanding of intervention delivery. Integration will follow an explanatory sequential approach, in which quantitative findings describing patterns of Fidelity, Dose, and Reach across sites are used to guide interpretation of qualitative data. Site-level quantitative data will be used to identify variation in intervention delivery (for example, differences in recruitment, protocol adherence, or dosing practices), and qualitative findings will be used to explain these patterns by exploring underlying contextual and behavioural influences.

A joint display will be used to merge qualitative and quantitative findings across constructs addressed by both datasets.^13^

Integration will be undertaken through comparison of findings across data sources, linking site-level delivery profiles with qualitative accounts of practice. This will enable identification of convergent, complementary, and divergent findings, and support development of explanatory accounts of how intervention delivery varied within and between sites.

Quality of Delivery will be derived as an integrative construct reflecting the combined influence of Context, Fidelity, Dose and Reach. Rather than being calculated as a formal composite score, Quality of Delivery will be derived through interpretation of integrated findings across data sources. This integrative analysis will enable exploration of how variation in intervention delivery may have influenced trial outcomes and will support interpretation of the main trial findings.

### Ethics and data management

The process evaluation forms part of the SINFONIA trial and has received approval from the relevant Research Ethics Committee alongside the main trial (East Midlands - Nottingham 2 Research Ethics Committee, 22/EM/0231, 20 December 2022).

Participation in the survey and interviews will be voluntary. Electronic informed consent will be obtained before participation in both the survey and interviews. For interviews, consent will be reconfirmed verbally immediately before data collection. All data will be anonymised before analysis and reporting. No individual participants, sites, or organisations will be identifiable in any publications. Data will be stored securely on institutional servers at Queen’s University Belfast and accessed only by authorised members of the process evaluation research team. Audio recordings of interviews will be deleted after transcription, and transcripts will be de-identified before analysis.

Given that the process evaluation explores implementation of clinical practice within a trial setting, findings will be reported in aggregate form to minimise the risk of identification of individual participants or sites.

## Discussion

### Reflexivity and rigour

The process evaluation is led by a multidisciplinary research team with complementary clinical and methodological expertise. The lead researcher (CD) is a clinician with a background in anaesthesia and experience of peri-operative practice. This provides valuable contextual insight into the clinical environment and intervention delivery but may also influence interpretation of the data.

To address this, reflexivity will be maintained throughout the research process, including awareness of how prior experience and assumptions may shape data collection and analysis. Analytical decisions and interpretations will be discussed within the wider process evaluation research team.

The process evaluation is conducted under the supervision of an experienced clinical trials methodologist (LE) with expertise in process evaluation and no direct involvement in anaesthetic practice. This provides an additional perspective and supports critical challenge of assumptions and interpretations.

Rigour will be enhanced through use of a systematic analytical approach, collaborative analysis, and iterative development of the analytical framework. Regular team discussions will be used to refine interpretations and ensure consistency across the analysis.

### Dissemination

Findings from the process evaluation will be disseminated through peer-reviewed publications and presentations at national and international conferences. Results will be reported alongside, and used to support interpretation of, the main SINFONIA trial outcomes.

## Authors' contributions

The process evaluation protocol was developed by all authors. CD led the preparation of the manuscript. All authors reviewed and contributed to the manuscript and approved the final version before submission.

## Ethics approval and consent to participate

The process evaluation forms part of the SINFONIA trial and has received approval from the relevant Research Ethics Committee alongside the main trial (East Midlands - Nottingham 2 Research Ethics Committee, 22/EM/0231, 20 December 2022). Electronic informed consent will be obtained before participation in both the survey and interviews. For interviews, consent will be reconfirmed verbally immediately before data collection.

## Consent for publication

Not applicable.

## Availability of data and material

Not applicable.

## Trial status

This manuscript reports SINFONIA protocol version number 4.0 of 02 October, 2024. Trial recruitment began in December 2022 and was completed in December 2025. Follow-up is ongoing and anticipated to complete in May 2026.

Process evaluation data collection commenced on 5 February 2025 and completed on 27 August 2025. Analysis is underway.

## Funding

The SINFONIA trial is funded by the National Institute for Health Research Health Technology Assessment programme: NIHR133056. CD is funded by Cardiff and Vale University Health Board and by Belfast Health and Social Care Trust.

## Declarations of interest

The authors declare that they have no conflicts of interest.

## References

[1] Moore G.F., Audrey S., Barker M. (2015). Process evaluation of complex interventions: Medical Research Council guidance. BMJ.

[2] Campbell N.C., Murray E., Darbyshire J. (2007). Designing and evaluating complex interventions to improve health care. BMJ.

[3] Lockwood I., Walker R.M., Latimer S., Chaboyer W., Cooke M., Gillespie B.M. (2022). Process evaluations undertaken alongside randomised controlled trials in the hospital setting: a scoping review. Contemp Clin Trials Commun.

[4] Barratt H., Campbell M., Moore L., Zwarenstein M., Bower P., Raine R., Fitzpatrick R., Barratt H. (2016). Health Serv Deliv Res.

[5] French C., Pinnock H., Forbes G., Skene I., Taylor S.J.C. (2020). Process evaluation within pragmatic randomised controlled trials: what is it, why is it done, and can we find it?-a systematic review. Trials.

[6] Silversides J.A., Savic L., Hiller L. (2025). Sugammadex or neostigmine for prevention of post-operative pulmonary complications after major abdominal or thoracic surgery: study protocol for the SINFONIA (sugammadex for preventioN of pOst-operative pulmonary complIcAtions) randomised controlled superiority trial. Trials.

[7] Cook D.S.D. (2023). StatPearls [Internet].

[8] Miskovic A., Lumb A.B. (2017). Postoperative pulmonary complications. Br J Anaesth.

[9] Vaghiri S., Prassas D., Krieg S., Knoefel W.T., Krieg A. (2023). The postoperative Effect of sugammadex versus acetylcholinesterase Inhibitors in Colorectal Surgery: an Updated Meta-Analysis. J Clin Med.

[10] Kletter M., Norman G., Dumville J., Wilson P. (2025). Process evaluations nested in randomised controlled trials of complex interventions: a scoping review of approaches and reporting. Trials.

[11] Emerson L.M., McAuley D.F., Blackwood B., Clarke M. (2025). The POETIC (PrOcess Evaluation of Trials In Critical care) Framework: a Structured Approach for Designing and Conducting Process Evaluations in Critical Care Trials. Crit Care Explor.

[12] Ritchie J., Spencer L., Bryman A., Burgess R.G. (1994).

[13] Fetters M.D., Tajima C. (2022). Joint displays of integrated data collection in mixed methods research. Int J Qual Methods.

